# Age group determines the acceptability of protein derived off-flavour

**DOI:** 10.1016/j.foodqual.2021.104212

**Published:** 2021-07

**Authors:** Sophie Lester, Leonardo Cornacchia, Camille Corbier, Katherine Hurst, Charfedinne Ayed, Moira A. Taylor, Ian Fisk

**Affiliations:** a Division of Food Nutrition and Dietetics, School of Biosciences, University of Nottingham, Sutton Bonington Campus, Loughborough LE12 5RD, United Kingdom; b National Institute for Health Research (NIHR) Nottingham Biomedical Research Centre, Division of Physiology, Pharmacology and Neuroscience, School of Life Sciences, University of Nottingham, Nottingham NG7 2UH, United Kingdom; c Danone Nutricia Research, Uppsalalaan 12, 3584 CT Utrecht, the Netherlands

**Keywords:** Healthy ageing, Protein, Flavour perception, Off-flavours, Older adults

## Abstract

Many older adults fail to meet their daily protein requirements, potentially due to social, physical and medical factors, including sensory and appetite changes. Additionally, our previous research has identified potential sulfurous off-flavours, originating from heat-treatment of protein ingredients, which could play a role in consumer acceptance. This study aims to determine the hedonic impact of these potential off-flavours when added to a dairy beverage, identify the specific off-flavour concentrations which cause rejection by consumers, and lastly investigate difference in acceptance between older and younger consumers. A rejection threshold (RjT) protocol was used, in combination with Best Estimate Thresholds (BET), whereby sulfurous flavours (dimethyl sulfide, dimethyl disulfide and dimethyl trisulfide), and diacetyl were added to create a range of concentrations. 95 participants (younger n = 49, 18–38 years; older n = 46, 60–79 years) tasted 7 pairs of samples (one blank and one with ascending off-flavour concentration) and selected their preferred samples. Sulfurous flavours negatively impacted consumer acceptance, however, the extent to which they impart a negative effect differs between age groups. Younger adults rejected samples containing low concentrations of sulfurous off-flavours (1.55 ppb), however, older adults rejected samples with concentrations over 3 times higher (5.08 ppb). When combined with sulfurous flavours, diacetyl increased the rejection threshold for both groups. In conclusion, these observations imply that a greater quantity of off-flavour may be present before acceptance is reduced in the older consumer group. Moreover, diacetyl demonstrates partial masking abilities of sulfurous off-flavours, and BET gave a more conservative estimate of acceptability. This knowledge will help guide sensory innovation of high-protein beverages for older consumers to support product acceptance and optimal intake.

## Highlights

• Heat-treatment of protein ingredients can impart sulfurous flavours into beverages.
• Sulfurous flavours negatively impacted consumer acceptance of a dairy beverage.
• Older adults had greater acceptance of sulfurous flavours compared to younger adults.
• Diacetyl reduced the negative impact of these compounds for both age groups.
• Best estimate thresholds give a conservative estimate of off-flavour acceptability.

## 1 Introduction

Daily protein recommendations for healthy adults range from 0.75 g protein/kg body weight/day in the United Kingdom (Department of Health, 1991) to 0.8 g protein/kg body weight/day in Europe and The United States (EFSA Panel on Dietetic Products Nutrition and Allergies, 2012). These recommendations are set irrespective of age, however, there is strong consensus amongst international bodies and researchers that daily protein requirements for healthy adults aged 65 years and above rise to 1.0–1.2 g protein/kg body weight/day (Bauer et al., 2013, Deutz et al., 2014). The increased requirement is due to an age-related resistance to the positive effects of dietary protein on body protein synthesis (known as anabolic resistance) along with a greater occurrence of disease-related protein catabolism (protein breakdown). In fact, if acute or chronic illness is experienced in older age, requirements are thought to rise further to 1.2–1.5 g protein/kg body weight/day (Deutz et al., 2014). The higher requirement could equate to a dietary protein increase of around 27 g protein a day for a typical 60 kg older adult, which is considerable.

The World Health Organisation defines malnutrition as deficiencies, excesses or imbalances in a person’s intake of energy, and/or nutrients (World Health Organisation, 2020). Two broad groups of malnutrition are identified: over-nutrition, such as in overweight, obesity and noncommunicable diseases such as heart disease and undernutrition, such as in stunting, wasting, underweight and micronutrient deficiencies (World Health Organisation, 2020). Protein-energy undernutrition (PEM), defined as an inadequate intake of energy and protein compared to requirements, is associated with delayed recovery from disease, poorer life quality and increased risk of morbidity and mortality (Leij-Halfwerk et al., 2019).

Many older adults fail to consume sufficient protein to meet their requirements (Ten Haaf et al., 2018), increasing their risk of muscle loss, sarcopenia and ultimately an increased risk of falls, fractures and hospital admissions (Bauer et al., 2013, Deutz et al., 2014, Lim et al., 2012).

The prevalence of undernutrition risk in the older population has been estimated to be 14%, and rises further to 21–35% for those living in institutions and care environments (Margetts et al., 2003, Schilp et al., 2012).

Currently in the UK, malnutrition is estimated to cost at least £23.5 billion; with older adults accounting for 52% of this cost (Stratton, Smith, & Gabe, 2018). To help combat undernutrition, the development of foods and beverages which are both nutritious and acceptable for older consumers, is an ongoing and crucial challenge for the food industry.

Factors inherent to the older consumer may generate challenges which limit the acceptability of high-protein foods and beverages. Older consumers experience oro-sensory changes which may alter their food sensory experience. Age-related reduction in gustatory sensitivity is known to occur in the older consumer group (Kälviäinen et al., 2003, Methven et al., 2012, Sergi et al., 2017) along with olfactory function (Ekström et al., 2019, Fluitman et al., 2019), which has found to be relatively more impeded by the ageing process (Stevens, Bartoshuk, & Cain, 1984). Olfactory impairments can contribute to altered food choices and reduced nutritional intake and status (Aschenbrenner et al., 2008, Duffy et al., 1995, Griep et al., 1995, Kremer et al., 2014, Somekawa et al., 2017).

Foods and beverages which are high in protein are particularly vulnerable to poor consumer acceptability as the protein molecules can be a source of undesirable sensory properties (Bull et al., 2017, Smith et al., 2016). Subjective mouth-feel sensations, such as mouth-drying and mouth-coating, can be caused by proteins (Bull et al., 2017, Withers et al., 2014) and are negative drivers of liking in dairy-based Oral Nutritional Supplement (ONS) (Thomas, Van Der Stelt, Prokop, Lawlor, & Schlich, 2016). Proteins may also impart new flavours to food and beverages, through interactions with other ingredients, degradation and/or processing induced chemical reactions (Al-Attabi et al., 2008, Cadwallader, 2016, Smith et al., 2016, Zabbia et al., 2012). Our previous research has identified sulfurous volatile flavour compounds in a commonly prescribed dairy-based ONS, some of which were rated as unpleasant by younger and older consumers (not yet published). Dimethyl sulfide (DMS), dimethyl disulfide (DMDS) and dimethyl trisulfide (DMTS) are a group of closely related sulfurous volatile flavour compounds, formed through Maillard reactions, from sulfurous essential amino acids, during high-temperature processing (Al-Attabi et al., 2008, Smith et al., 2016, Zabbia et al., 2012). The pungent character and high-impact of these flavour compounds means they contribute to the cooked, heated and sulfurous flavour notes in thermally processed milk (Al-Attabi et al., 2008, Vazquez-Landaverde et al., 2006).

Another flavour compound of interest, which often occurs concurrently with sulfides in dairy foods (Zabbia et al., 2012), is the volatile flavour compound diacetyl (2,3-butandione). Diacetyl is noted for its appealing butter-like aroma and flavour (Antinone et al., 1994, Clark and Winter, 2015), and can be present naturally in many dairy products such as butter and cheese (Clark & Winter, 2015). In common with sulfides, diacetyl can be formed through Maillard reactions during thermal treatment of dairy products (Zabbia et al., 2012). Previous studies investigating the hedonic impact of diacetyl in dairy products have found that sour creams with the greatest perceivable intensities of diacetyl had the greatest consumer acceptability, compared to sour creams with lower perceivable intensities of diacetyl (Shepard, Miracle, Leksrisompong, & Drake, 2013). Antinone et al (1994) found an increase in liking for attributes of cottage cheese as a function of diacetyl concentration, with the mean flavour score peaking at 1000 ppb. Drake et al (2009) identified diacetyl flavour to be a driver of liking in full-fat cottage cheese. It is not yet known how the combination of sulfurous flavours and diacetyl affect the acceptability of foods and beverages. This study firstly aimed to examine the hedonic impact (positive, negative or no impact) of potential off-flavour compounds when added in increasing concentrations to a flavoured dairy beverage. Secondly, we aimed to identify the concentrations at which rejection occurred by consumers (the rejection threshold). Thirdly, by comparing rejection threshold concentrations for each age group, we investigated whether human age was a factor influencing consumer acceptance of these flavours. Lastly, we aimed to compare suitability of two separate rejection threshold methodologies (graphical approach (RjT_50_) and Best Estimate Thresholds (BET) and the impact of each on our conclusions. Flavour compounds were studied both alone, and in combination, to ensure any flavour-interactions were captured.

## 2 Materials and methods

This study was approved by Faculty of Medicine and Health Sciences Research Ethics Committee at the University of Nottingham (Reference No. 156-1810).

### 2.1 Participants

Forty-nine younger participants and forty-six older participants were recruited to take part in the study from The University of Nottingham and local villages via an email invitation and poster advertisements. Inclusion criteria were: age between 18 and 40 years or 60–80 years, male or female and smokers or non-smokers. These age ranges were chosen to incorporate a large range with a defined age gap between the younger and older group. The World Health Organisation has previously defined older age as 60 years and older (Mathers, Stevens, Boerma, White, & Tobias, 2015). 80 years was chosen as an upper age limit to minimise risk of harm due to increasing prevalence of frailty with age. Exclusion criteria were: food allergies or intolerance to dairy (or other ingredients used in the beverage), pregnancy or breastfeeding, or known sensory impairments (unrelated to ageing) in taste or smell. A questionnaire was used to collect health, lifestyle, and demographic information, including habitual milk consumption, and confirmed their eligibility to take part (this data can be found in Table 1). Informed consent was collected from all participants, but participants were not informed that the study was investigating differences in flavour.

**Table 1:** Health and demographic information, and milk beverage consumption behaviour and preference, for the younger and older groups of participants included in the study.

	Younger	Older
**Health and demographic information**		
n	49	46
Mean age in years (range)	23 (18–38)	69 (60–79)
Male	24.5	39.1 18: 28
Female (%)	75.5	60.9
Mean no. regular medication taken by each participant (daily)	0.3	2.3
Percentage of participants with chronic health condition	6	20
Percentage of participants currently regularly smoking	2	0
Percentage of participants who previously regularly smoked (>5 years)	6	33
Percentage of participants with previous experience in food sensory analysis	57	37
**Milk consumption behaviour and preference**		
Percentage preferring pasteurised milk	90	91
Percentage preferring UHT milk	4	9
Percentage preferring dairy alternatives	6	0
Percentage who find the flavour of UHT milk enjoyable	49	52
Percentage regularly consuming UHT milk (once a month or more)	47	65
Regularly consume flavoured dairy beverages (milkshakes)	80	41
Enjoy banana as a flavour	88	98

### 2.2 Materials

Pasteurised whole milk was purchased from a national supermarket in the UK. It was essential to use milk that had only undergone a gentle heat-treatment such as pasteurisation, rather than Ultra High Temperature (UHT), in order to limit the presence of heat-associated flavours. Each bottle of milk also had the same production date. Maltodextrin (DE 19) and banana flavourings were gifted by Danone Nutricia Research®, NL. Food-grade diacetyl (2,3-butandione) was supplied by De Monchy Aromatics Ltd®, UK, and food-grade dimethyl sulfide (DMS), dimethyl disulfide (DMDS) and dimethyl trisulfide (DMTS) were purchased from Sigma-Aldrich®, US.

### 2.3 Dairy beverage preparation

The banana flavoured dairy beverage was produced in a single batch to limit batch-to-batch variations in flavour. Banana flavourings (0.05 g/L) and maltodextrin (300 g/L) were incorporated into the milk by electric hand mixing at room temperature (20 °C ± 1). The beverage was separated into 4 L milk bottles and stored frozen (−18 °C) until the evening before a study day when the desired quantity was defrosted in a refrigerator (3 °C) overnight. The beverage was stored for no longer than 3 weeks. No perceivable changes in flavour occurred during this time and no separation was observed.

On the morning before a study session, flavour compounds were ‘spiked’ into the beverage to create the desired concentrations (Table 2). Propylene glycol (PG) was used as the flavour carrier and the same volume of PG was also spiked into blank samples to ensure matrix uniformity between blank samples and flavour ‘spiked’ samples. The concentrations of flavours used were determined by concentrations previously quantified in a commercial product used as a reference (data not shown).

**Table 2:** Flavour concentrations (ppb) used in the rejection threshold experiments.

Level	Experiment 1: Sulfurous flavours				Experiment 2: Diacetyl	Experiment 3: Mixture	Equivalent concentration in commercial product
	DMS	DMDS	DMTS	Total			
1	0	0	0	0	0	0	0%
2	0.18	0.71	0.18	1.07	42	43.07	33%
3	0.37	1.42	0.36	2.15	85	87.15	66%
4	0.56	2.13	0.54	3.23	128	131.23	~100%
5	0.75	2.84	0.72	4.31	171	175.31	133%
6	0.94	3.55	0.90	5.39	214	219.39	166%
7	1.13	4.26	1.08	6.47	257	263.47	~200%

For Experiment 1, three closely related sulfide compounds (DMS, DMDS, DMTS) termed the common name ‘sulfurous flavours', were spiked into the beverage. For Experiment 2, diacetyl alone was spiked into the beverage. For Experiment 3, both sulfurous flavours and diacetyl were spiked into the beverage, in the same concentrations used in the previous experiments. Once the concentrations were prepared, the beverage was kept refrigerated until being pipetted into individual 10 mL samples.

For all experiments, the first concentration was chosen to be 0 ppb, in an effort to obtain a RjT closer to 50% (assuming that there would be an equal chance of participants choosing either of two samples) (See Section 1.5 for statistical methods). Following this, concentrations of sulfurous flavours and diacetyl increased by increments of 33% of the concentration quantified in the commercial product (Level 4, Table 2). This increment in flavour concentration is smaller than those used in previous rejection threshold studies (for example, Prescott et al (2005) increased concentrations of TCA by a factor of 2, or 100%). In the present study, this smaller concentration increment was chosen because the high impact of sulfurous flavours was known prior to the experiment.

### 2.4 Protocol on study days

All sensory testing took part in The University of Nottingham’s Sensory Science Centre (Sutton Bonington Campus) in sensory booths designed to ISO standards (ISO8589:1988). Study sessions were mixed with both older and younger participants and participants attended 1 session per week in a randomised order (each session consisting of Experiments 1, 2 or 3). Participants were instructed not to wear strong smelling cosmetics and not to eat, drink or smoke 2 h before attending a session.

In each study session, a rejection threshold design was employed whereby participants were provided with a series of 7 paired preference tests (each pair containing one blank sample, and one sample containing an ascending concentration of off-flavour). Paired preference tests have been recognised as an appropriate sensory test for use with older adults due to their relative simplicity (Methven, Jiménez-Pranteda, & Lawlor, 2016). Each sample was 10 mL in volume and served in 30 mL plastic cups, each labelled with a random 3-digit code. Samples were served chilled, in-line with the typical serving temperature for flavoured milkshake beverages. The order of presentation within a pair was randomised.

Participants were instructed to taste each sample within a pair, from left to right, and then were asked the question “Which sample do you prefer?”. They were instructed to indicate their response by selecting the sample code. A ‘no preference’ option was not provided. To record their responses, participants were given the choice to use a computer or a paper copy of the same test, of which 2 older and 1 younger participant chose to use paper. Online data was collected using Compusense Cloud® (Compusense, Ontario, Canada). In-between tasting each sample within a pair, participants were asked to rinse their mouth with water (Evian, Danone, France). In-between tasting of pairs, during a compulsory 2-minute break, participants were asked to cleanse their palate by chewing and swallowing one pre-prepared slice of green apple (Golden Delicious, Tesco, UK), and rinsing their mouth with water.

To gain an insight into the reasons for rejection, participants were provided with an open-ended question after each pair, where they were asked “Why did you choose this sample as your preferred sample?” and allowed to write freely.

### 2.5 Statistical analysis

All statistical analysis was conducted using the software XLSTAT® statistical and data analysis solution (version 20.6.01, Addinsoft, Long Island, NY, USA) or GraphPad Prism® (version 7.0, San Diego, CA, USA).

Constant values of + 2 (sulfurous flavours) or + 100 (diacetyl and mixture) were added to the concentrations (ppb), to omit zero values and enable the statistical analysis. For example, at the first concentration of diacetyl (0 ppb), a constant value of 100 was added, to give a final value of 100 ppb. At the second concentration of diacetyl (42 ppb), a constant value of 100 ppb was added, to give a final value of 142 ppb. These constant values were later subtracted (as described below).

At each concentration level, the percentage of participants preferring the blank sample in a pair (hence rejecting the ‘spiked’ sample in a pair), was plotted on the y-axis with the log concentration on the x-axis. The hedonic impact was thus indicated by consumer preference, relative to the blank, for the spiked sample across increasing concentrations of the flavour compounds of interest.

To calculate rejection thresholds using a graphical approach, a sigmoidal variable slope dose–response function was fitted through the data points, using the Hill equation. The Hill equation, commonly used in pharmacology, describes four parameters: the top of the curve (max), the bottom of the curve (min), the spot halfway between min and max (EC_50_ or RjT_50_) and the slope of the curve (the Hill coefficient). The 2-AFC chance corrected probability (75% rejection) gave the rejection threshold (LogRjT_50_) and this was automatically calculated by GraphPad Prism® (the LogEC_50_ value, see Harwood et al (2012) for a concise description of statistical methods). To obtain the RjT_50_ concentration (ppb), the antilog of ‘LogRjT_50_′ is found and the constant values subtracted. Due to absence of data points at 50% (chance) for some of the investigations, some RjT_50_ values were ambiguous. Therefore, two of the four parameters within the Hill equation (the minimum and maximum) were constrained at values of 50 and 100, as described in Harwood et al (2012), and the curve was re-fit. This constraint was applied to all investigations in order to compare accurately between them.

It has recently been recommended that when estimating consumer RjT, both a graphical approach (described above) and a Best Estimate Threshold (BET) approach should be utilised (Murray et al., 2019). Thus, for a complementary comparison between age groups, BET were also calculated by using the adapted method presented by Murray et al (2019) by taking the geometric mean of the first concentration whereby individual participants preferred the blank, and the next lowest concentration where participants preferred the spiked sample. The geometric mean of individual BET within a group gave the age-group BET. Due to uneven distribution of this data, the non-parametric test Mann-Whitney U was used to statistically compare group values.

Qualitative data was interpreted by a method based on Ares et al., 2008, Ares and Deliza, 2010. Descriptive reasons consumers gave for rejecting the flavour spiked sample, at the concentration level immediately following the group rejection threshold (RjT_50_), were compiled for both age groups. To generate categories, three researchers independently searched the data for recurrent and similar terms. Both personal interpretation and synonyms (as determined by an English dictionary) were employed to classify terms into categories. After the independent analysis, a meeting between the researchers resulted in consensus on the categories. For ease of interpretation by the reader, categories were further categorised into sensory modalities. Within each age-group, category frequencies were determined by counting the number of individual consumers who mention each category. The percentage of consumers who mentioned each category, out of the number of consumers who rejected at this level (within each age-group), was calculated. Only categories which were mentioned by > 5% of consumers are shown.

## 3 Results

### 3.1 Hedonic effect and rejection thresholds determined by a graphical approach (RjT_50_)

#### 3.1.1 Experiment 1: Sulfurous flavours

As the concentration of sulfurous flavours increased, higher percentages of participants preferred the blank samples, signifying that sulfurous flavours had a negative hedonic impact on consumer acceptance of the dairy beverage. This was true for both age groups, however, there were differences in the concentration at which rejection (75% rejection) occurred. When combined into a single group, the group rejection threshold (RjT_50_) was 2.47 ppb, however, when participants were separated into the respective age categories, younger adults reached rejection at 1.55 ppb and older adults reached rejection at 5.08 ppb (over 3 times higher). Importantly, unlike older adults, younger adults rejected sulfurous flavours at a concentration lower than the concentration in the commercial product (3.23 ppb, Level 4).

##### 3.1.1.1 Qualitative reasons for rejection of sulfurous flavour-spiked sample

At the closest level to the RjT_50_ (concentration level 3), the main reasons given by the younger group for rejection of samples with sulfurous flavour included detection of ‘Off-flavour’ (18%) and ‘Unpleasant aftertaste’ (15%). In contrast to this, at the closest level to the RjT_50_ (concentration level 6) no older adults (0%) gave these as reasons for rejecting the samples. The main reasons given by the older adults to reject the samples with sulfurous flavour were ‘Unpleasant aroma’ (7%) and ‘Weaker flavour’ (7%). Older adults stated positive reasons for accepting the blank sample more frequently, such as ‘pleasant aroma or ‘good banana flavour’, rather than explicitly state negative reasons for rejecting the sample containing sulfurous flavour.

#### 3.1.2 Experiment 2: Diacetyl

Over increasing concentrations of diacetyl, only marginally greater percentages of participants chose the blank samples over the flavour spiked samples, indicating a small negative hedonic impact. Although younger adults demonstrated greater rejection than the older adults, neither age group reached 75% rejection. The Hill equation predicted that if concentrations of diacetyl did continue to increase rejection would have occurred at 592 ppb for younger adults and 1738 ppb for older adults (LogRjT_50_ values are 2.84 and 3.24 respectively). Qualitative data is not shown as a rejection threshold was not reached.

#### 3.1.3 Experiment 3: Mixture of sulfurous flavours with diacetyl

When the sulfurous flavours were combined with diacetyl, in a new series of paired preference tests (Experiment 3), we see that 75% rejection (RjT_50_) is reached. The added flavours had a negative hedonic impact on consumer acceptance of this dairy beverage: as the concentration off ‘off-flavours’ increased, a greater number of consumers preferred the blank samples. If considering the participants as a single group, the rejection threshold occurred at 188 ppb. However, if again separated into their respective age categories, younger adults reached 75% rejection at 163 ppb and older adults reached rejection at a higher value of 263 ppb. For both age-groups, when compared to Experiment 1 (sulfurous flavour alone), the point at which RjT_50_ occurred increased, with rejection occurring at higher concentration levels.

##### 3.1.3.1 Mixture: Qualitative reasons for rejection of flavour-spiked sample

At the closest level to the RjT (concentration level 5), the main reasons given by younger adults for rejecting the samples containing both sulfurous flavours and diacetyl included detection of ‘Off-flavour’ (20%) and ‘Unpleasant aroma’ (19%). In line with Experiment 1 (Section 2.1.1.1), ‘Unpleasant aftertaste’ (13%) was also a reason given frequently by younger consumers. In contrast, lower percentages of older adults gave detection of ‘Off-flavour’ (2.5%) and ‘Unpleasant aftertaste’ (0%) as reasons for rejecting the spiked samples. In agreement with the data from Experiment 1 (Section 2.1.1.1), at the closest level to the RjT (concentration level 7), older adults stated a ‘Weaker flavour’ (10%) and ‘Unpleasant aroma’ (8.9%) as the main reasons for rejecting samples containing sulfurous flavour and diacetyl. In comparison, less younger adults stated a ‘Weaker flavour’ as a reason for rejecting the samples containing sulfurous flavour and diacetyl (7%). In agreement with Experiment 1, older adults stated positive reasons for accepting the blank sample more frequently, rather than state negative reasons for rejecting the sample containing off-flavours.

### 3.2 Rejection thresholds as determined by Best estimate thresholds (BET)

Best Estimate Thresholds (BET) were also calculated for each experiment (Fig. 4). For Experiment 1 (sulfurous flavours alone), younger adults had significantly lower BET than the older adults (p = 0.0009). For Experiment 2 (diacetyl), although younger adults had lower thresholds, there is no significant difference between the age-groups. When sulfurous flavours and diacetyl was again combined in Experiment 3, there is a significant difference between the age groups (p = 0.0269).

### 3.3 Comparison between graphical approach (RjT_50_) and Best estimate thresholds (BET)

For both age-groups, all calculated BET values were lower than the threshold values calculated by a graphical approach (RjT_50_) (Fig. 5). This discrepancy between the methodologies led to important differences. For example, for Experiment 1 (sulfurous flavour), older adults had RjT_50_ values higher than the concentration in product. This finding contrasts with the BET values for this age group, which were below the concentration in product.

## 4 Discussion

This study firstly aimed to examine the hedonic impact of potential off-flavour compounds, when added in increasing concentrations to a dairy beverage. Secondly, we aimed to identify the concentration at which consumer rejection occurred and lastly, whether human age was a factor influencing consumer acceptance of these flavours. Flavour compounds were studied alone, and in combination, to ensure any flavour-interactions were captured.

**Fig. 1:**
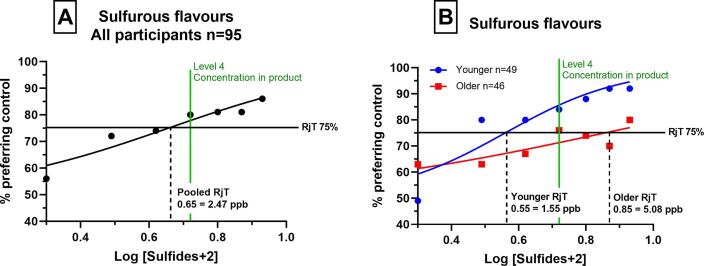
Consumer rejection thresholds of sulfurous flavours (experiment 1) as determined by a graphical approach (RjT_50_). Proportion preferring the blank (y-axis) plotted against the log concentration of flavourings (x-axis), which were spiked into the beverage. A) shows global consumer rejection of all ages (n = 95), B) shows comparison of younger consumer (blue, n = 49) and older consumers (red, n = 46). (For interpretation of the references to colour in this figure legend, the reader is referred to the web version of this article.)

Sulfurous flavours can impart essential flavours and background notes to some foods and beverages, such as vegetables and coffee (Al-Attabi et al., 2008, Buttery et al., 1976, Kim et al., 2018, Mishra et al., 2017). However, in other products, or at certain concentrations, they may become undesirable (Zabbia et al., 2012). In the current study, for both age groups, as the concentration of sulfurous flavours increased in the dairy beverage, a greater percentage of consumers preferred the blank samples (Fig. 1). This means that sulfurous flavours had a negative hedonic impact on consumer acceptance of the dairy beverage.

However, we observed that the impact was greatly dependent on age-group, as the rejection threshold occurred at much lower concentrations for younger adults, compared with older adults. Younger adults rejected samples at almost the lowest concentration of sulfurous flavour (Fig. 1). This demonstrates that younger consumer acceptance of the dairy beverage was affected strongly by the presence of sulfurous flavours, even at low concentrations. In comparison, older adults rejected samples at higher concentrations, demonstrating that sulfurous flavours have a less strong negative impact on older consumer acceptance of the dairy beverage. Best estimate thresholds (BET) were also significantly different between age groups for sulfurous flavours (p = 0.0009) and for sulfurous flavours in combination with diacetyl (mixture) (p = 0.0269).

This age-related difference in consumer acceptance could be attributed to either i) sulfurous flavours are perceived more pleasantly or ii) older adults have an impaired ability to detect sulfurous flavours. There is some evidence to suggest that sulfurous flavour compounds become less unpleasant with human ageing (Wysocki & Gilbert, 1989). In addition, we hypothesise that impairments in olfactory sensitivity played a substantial role in the findings. This hypothesis is supported by a vast amount of research evidencing that olfactory ability decreases with ageing due to age-related alterations within the nose, olfactory epithelium, bulb, and higher brain structures (Doty & Kamath, 2014). In addition, many medications used to treat age-related conditions, such as hypertension, are known to alter taste and smell acuity (Schiffman & Zervakis, 2002). In the current study, the older adult group reported taking almost 8 times higher amounts of daily medication in comparison to the younger group (Table 1). Age-related impairments in taste and smell are known to affect older adults ability to perceive flavour and have a negative influence on older adults dietary behaviour, nutritional intake and nutritional status (Aschenbrenner et al., 2008, Duffy et al., 1995, Griep et al., 1995, Kremer et al., 2014, Somekawa et al., 2017). Age-related sensory impairments are often debilitating. Though, this current research is a unique example of how age-related changes may also offer benefits to the older consumer as beverages which have high or enhanced nutritional value, but undergo inevitable sensory changes, maintain greater acceptability. This current finding supports the hypothesis of Mattes (2012) who suggested that age-related sensory losses may diminish detection of undesirable flavour notes, thus promoting intake in older populations who are often presented with novel foods for therapeutic nutritional reasons. Our observations are also supported by previous research which found that older adults, particularly those with poor olfactory abilities, were more willing to accept novel foods with unpleasant odours than younger subjects (Pelchat, 2000).

In contrast, acceptance by younger consumers was reduced greatly by sulfurous flavours, even at low concentrations. Currently, many younger consumers aim to increase their protein intake for health or athletic reasons (Hartmann and Siegrist, 2016, Sung and Choi, 2018, Whitehouse and Lawlis, 2017). Dairy-protein ingredients such as dry powder concentrates, or ready to drink high-protein beverages, are a popular choice for many (Singh, 2020, Singh et al., 2019). Thermal treatment is frequently used to prolong shelf life and ensure consumer safety of these high-protein products (Singh, 2020) but this can result in the formation of undesirable flavours (Al-Attabi et al., 2008, Cadwallader, 2016, Zabbia et al., 2012). It has previously been found that consumers are unlikely to compromise on taste for positive health outcomes (Verbeke, 2006). It is thus important that high-protein products deliver both palatable flavour and nutritious ingredients to ensure consumer satisfaction. The source of off-flavours, such as amino acids, are often essential nutrients which manufacturers cannot remove from a product. Flavour masking should therefore be prioritised, along with changes to processing conditions, to ‘hide’ or reduce formation of off-flavours whilst maintaining microbiological stability (Cadwallader, 2016).

Our observations demonstrate that diacetyl partially masked the undesirable effects caused by sulfurous flavours. This was observed for both age-groups as the combined effect of sulfurous flavours with diacetyl increased the point at which sulfurous flavours became objectionable (Fig. 3). This finding demonstrates the importance of flavour interactions and may occur via a ‘mixture suppression’ mechanism, whereby the perceived intensity of an odorant mixture is less than that of the individual components (Cadwallader, 2016). When investigating off-flavours, future researchers should consider the combined effect of all aroma-active compounds which contribute to a flavour.

**Fig. 2:**
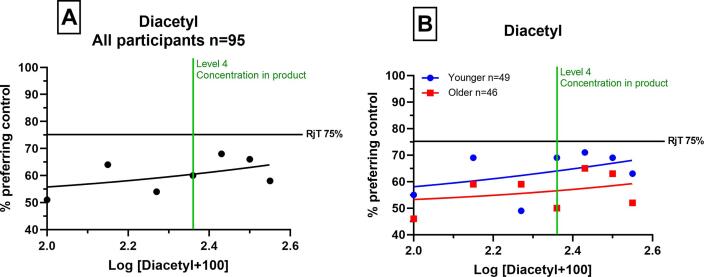
Consumer rejection thresholds of diacetyl (experiment 2) as determined by a graphical approach (RjT_50_). Proportion preferring the blank (y-axis) plotted against the log concentration of flavourings (x-axis), which were spiked into the beverage. A shows global consumer rejection of all ages (n = 95), B shows comparison of younger consumer (blue, n = 49) and older consumer (red, n = 46) groups. (For interpretation of the references to colour in this figure legend, the reader is referred to the web version of this article.)

**Fig. 3:**
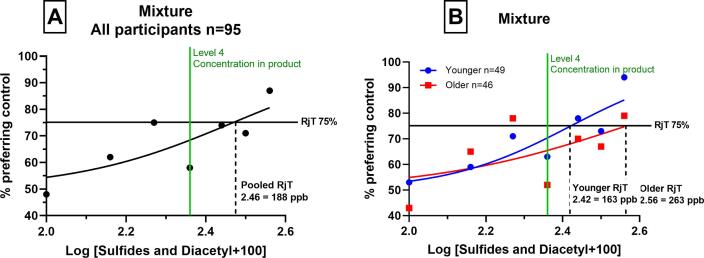
Consumer rejection thresholds of sulfurous flavours and diacetyl combined in a mixture (experiment 3) as determined by a graphical approach (RjT_50_). Proportion preferring the blank (y-axis) plotted against the log concentration of flavourings (x-axis), which were spiked into the beverage. A shows global consumer rejection of all ages (n = 95), B shows comparison of younger consumer (blue, n = 49) and older consumer (red, n = 46) groups. (For interpretation of the references to colour in this figure legend, the reader is referred to the web version of this article.)

When diacetyl was added to the beverage alone (Experiment 2, Fig. 2), 75% rejection was not reached for either age-group, meaning diacetyl cannot be considered an off-flavour in the beverage at the concentrations studied. Previous findings that diacetyl increased the acceptance of dairy products (Antinone et al., 1994, Drake et al., 2009, Shepard et al., 2013), for example in cottage cheese at concentrations of 1000 ppb (Antinone et al., 1994), are not supported by the current study. The concentration increments used in this present study were small (33%) and therefore the objectionable concentration was not reached. The Hill equation predicted that consumer rejection would have occurred at concentrations of 592 ppb for younger adults and 1,738 ppb for older adults. These higher concentrations of diacetyl can occur in food and beverages; concentrations as high as 27,000 ppb have been reported in dairy products such as yoghurt (Clark & Winter, 2015) but the hedonic effects are likely to be product and matrix dependent.

The statistical methodology chosen to calculate rejection thresholds is important. Murray et al (2019) recently recommended that a complementary approach encompassing both RjT_50_ and BET methodologies would be beneficial when estimating the acceptability of sensory properties. We observed that all rejection thresholds calculated by the BET approach were lower than those concentrations calculated using the graphical approach (RjT_50_) (See Fig. 5). Therefore, in agreement with Murray et al (2019), BET are a conservative approach to determine acceptability. It may sometimes be more appropriate to use the BET methodology for products where acceptance is particularly important. For example, Foods for Special Medicinal Purposes (FSMPs), such as oral nutritional supplements, are typically prescribed to patients and not purchased out of ‘desire’ like most products. Subsequently, acceptability is particularly important for sufficient intake.

**Fig. 4:**
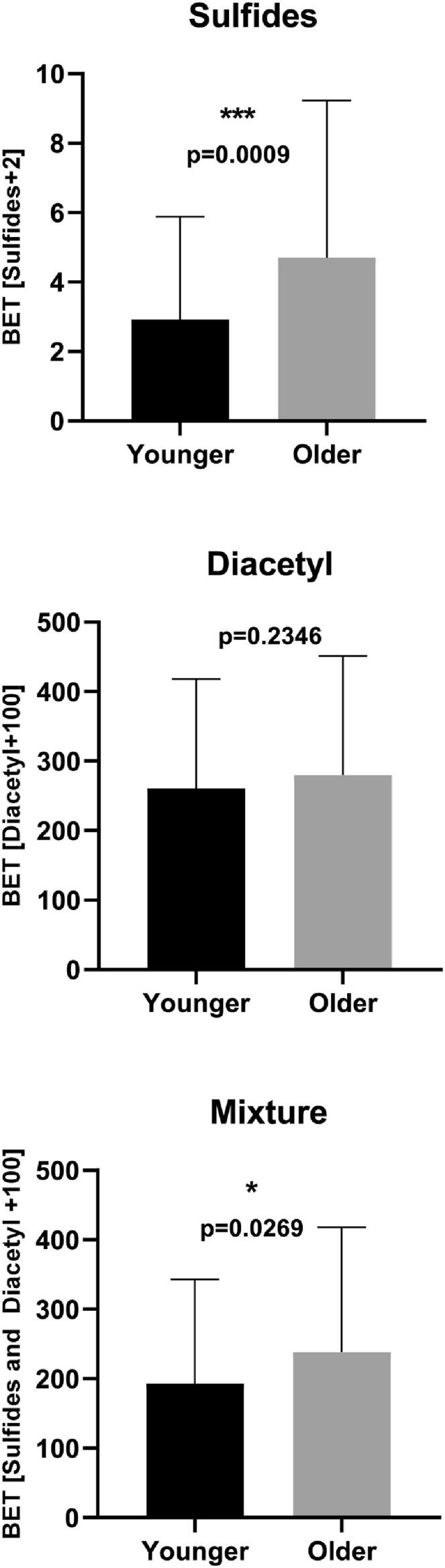
Best estimate thresholds (BET) of younger consumers (black) and older consumers (grey) for experiments 1–3. Values show geometric mean ± geometric standard deviation. Differences between groups were analysed by Mann Whitney U (p = 0.05). * indicates p ≤ 0.05, ** indicates p ≤ 0.01, *** indicates p ≤ 0.001.

**Fig. 5:**
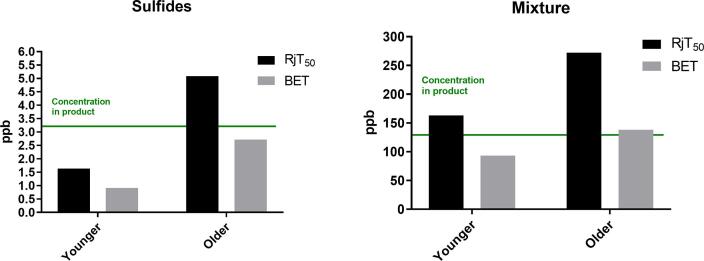
Comparison of the Rj_50_ (black) and BET (grey) values for both age groups. Left shows Experiment 1 (sulfurous flavours) and right shows Experiment 3 (mixture). Experiment 3 (diacetyl) was not included in this comparison because a RjT_50_ value was not reached for this flavour compound.

Younger adults stated ‘Off-flavour’ and ‘Unpleasant aftertaste’ as main reasons for rejecting samples containing sulfurous flavours. In contrast, very few older adults cited these as reasons for rejection (Table 3 and Table 4). Older adults may be less aware of unpleasant flavours lingering post-consumption, or perhaps due to reduced sensory acuity, older adults are less able to articulate specific sensory effects caused by off-flavours. Though, older adults are more vulnerable to fatigue from multiple testing, along with difficulties writing and expressing themselves (Methven et al., 2016). Nevertheless, older adults were more inclined to state positive reasons for preferring the blank samples so perhaps reluctance to cause offence to the researchers contributed to the differences between age-groups. To mitigate this, future research could ask “What did you *dislike* about the rejected sample?”, however this approach could make the consumers aware of undesirable sensory properties which otherwise they may not have noted. It is also worth discussing that 10% more older adults stated they ‘Enjoyed banana as a flavour’ compared to younger adults (Table 1), which may have driven a more positive sensory experience overall and increased their inclination to state positive reasons for choosing the control samples over the off-flavour samples. Multimodal effects of sulfurous flavours were also observed: a number of participants reported perceived textural reasons for preferring the blank samples, such as ‘Less creamy’ (Table 3) and ‘Watery mouthfeel’ (Table 4). A number of participants also reported sweetness as reasons for preferring the blank samples, such as ‘Less sweet’ (Table 3). These reasons were stated despite the blank and ‘spiked’ samples having identical matrices and levels of macronutrients. It is known that volatile flavour compounds can influence the perception of both texture and sweetness. For example Saint-Eve et al (2004) found that yoghurts containing fatty flavours were perceived as thicker, whereas a mixture of flavours were perceived as less thick. Aroma-taste interactions have also been found, for example, Saint-Eve (2004) found that yoghurts with the same sucrose content were perceived to be sweeter when flavoured with strawberry flavours. To the authors knowledge the reported effects of sulfurous flavours on tastant perception (sweetness) and texture perception have not been reported previously.

**Table 3:** Frequencies (Freq), and percentages (%) of consumers who gave reasons within each category as a reason for rejecting the sample containing sulfurous flavour, counted at the concentration level immediately following the point of rejection for each age-group. % are the proportion of consumers who rejected at the specific concentration level.

Category	Examples	Older Concentration level 6		Younger Concentration level 3	
		Freq	%	Freq	%
**Flavour and taste**					
Unpleasant flavour	Less pleasant taste, Bad taste	2	3	6	8
Off-flavour	Metallic taste, sour taste, oniony taste	0	0	14	18
Stronger flavour	Less subtle taste, Full flavour, Too much banana flavour	2	3	4	5
Weaker flavour	Weak banana taste, Less flavour, Less strong	5	7	6	8
Less sweet	Not sweet enough, Sample was less sweet	1	1	7	9
**Aroma**					
Unpleasant aroma	Less pleasant aroma	5	7	7	9
Specific off-aroma	Bitter aroma, Sour aroma, Rancid smell	1	1	8	10
**Texture**					
Thinner texture	Watery texture, Less creamy, Less thick	2	3	5	6
**Aftertaste**					
Unpleasant aftertaste	Weird aftertaste, Tangy aftertaste, Less pleasant aftertaste	0	0	12	15

**Table 4:** Frequencies (Freq), and percentages (%) of consumers who gave reasons within each category as a reason for rejecting the sample contaiing sulfurous flavours and diacetyl, counted at the concentration level immediately following the point of rejection for each age-group. % are the proportion of consumers who rejected at the specific concentration level.

Category	Examples	Older Concentration level 7		Younger Concentration level 5	
		Freq	%	Freq	%
**General**					
Unpleasant	Less palatable, Less pleasant, Unpleasant	1	1	9	13
**Flavour and taste**					
Unpleasant flavour	Less pleasant flavour, Unpleasant taste	3	4	8	11
Off-flavour	Metallic taste, Sour taste, Less fresh taste	2	3	14	20
Stronger flavour	More potent flavour, Less subtle, More strong flavour	1	1	5	7
Weaker flavour	Weaker milk taste, Less strong taste, More watery taste	8	10	5	7
Less sweet	Less sweet	1	1	4	6
Artificial flavour	More synthetic flavour, More artificial taste	0	0	4	6
**Aroma**					
Unpleasant aroma	Off-putting aroma, Less pleasant smell	7	9	13	19
**Texture**					
Thinner texture	Watery mouthfeel, Less creamy texture	2	3	4	6
**Aftertaste**					
Unpleasant aftertaste	Nasty aftertaste, Less pleasant after taste, Weird aftertaste	0	0	9	13
Stronger aftertaste	More aftertaste, More strong aftertaste	1	1	5	7

### 4.1 Strengths and limitations of research

To confirm our hypothesis that the higher rejection thresholds in the older adult group were driven by lower olfactory abilities it would have been advantageous to measure olfactory sensitivity alongside the rejection thresholds, for example through detection threshold testing. A strength of the study was the high participant compliance, which was 100% for both age groups. To complete the additional olfactory sensitivity investigation, a greater number of samples would have been required. This would have increased the risk of inducing fatigue in participants (of which, older adults are more vulnerable) but a greater number of study visits may have reduced participant compliance.

## 5 Conclusions

To support worldwide healthy ageing, the development of nutritious and acceptable high-protein foods and beverages is crucial. This study found that protein-originating sulfurous flavours negatively influenced consumer acceptance of a banana flavoured dairy beverage. The extent to which sulfurous flavours had a negative effect differed by age group. Compared with younger adults, sulfurous flavours were more acceptable for older adults, which was likely to have been driven by age-related impairments in sensory perception. This age-related effect may be a benefit to the older consumer, by increasing their willingness to accept protein fortified beverages, thus promoting nutritional intake. As a further finding, irrespective of age, the addition of diacetyl increased the concentration at which rejection occurred, subsequently providing masking benefits. This partial masking capability of diacetyl may be a solution to improve the palatability of beverages, a finding particularly relevant for younger consumers who were relatively less accepting of sulfurous off-flavours. Due to our findings, we recommend testing the acceptability of sensory properties with the consumer age-group of interest. In addition, we propose that BET is a more appropriate method to estimate consumer acceptance of nutritional food and beverage products where acceptability is essential for sufficient intake.

## Author contributions

All authors approved the final version of this article. Sophie Lester carried out the data collection. Sophie Lester, Ian Fisk, Moira Taylor, Leonardo Cornacchia, Katherine Hurst, Charfedinne Ayed and Camille Corbier contributed to the conceptualisation, design of the project, interpretation of results and preparation of the manuscript.

## Declaration of Competing Interest

The authors declare that they have no known competing financial interests or personal relationships that could have appeared to influence the work reported in this paper.

## Figures and Tables

**Table:** 

Age-group	Chronic health conditions	Percentage of group with chronic health condition
Younger	Iron deficiency, Adrenal abnormality, Asthma.	6
Older	Chron’s disease (2), Undisclosed (2), Arthritis, Thrombocythemia, Heart disease, Hypertension, Asthma, Bronchitis.	20
